# The K46 and K5 capsular polysaccharides produced by *Acinetobacter baumannii* NIPH 329 and SDF have related structures and the side-chain non-ulosonic acids are 4-O-acetylated by phage-encoded O-acetyltransferases

**DOI:** 10.1371/journal.pone.0218461

**Published:** 2019-06-20

**Authors:** Johanna J. Kenyon, Nikolay P. Arbatsky, Mikhail M. Shneider, Anastasiya V. Popova, Andrei S. Dmitrenok, Anastasiya A. Kasimova, Alexander S. Shashkov, Ruth M. Hall, Yuriy A. Knirel

**Affiliations:** 1 Institute of Health and Biomedical Innovation, School of Biomedical Sciences, Faculty of Health, Queensland University of Technology, Brisbane, Australia; 2 N. D. Zelinsky Institute of Organic Chemistry, Russian Academy of Sciences, Moscow, Russia; 3 M. M. Shemyakin & Y. A. Ovchinnikov Institute of Bioorganic Chemistry, Russian Academy of Sciences, Moscow, Russia; 4 Moscow Institute of Physics and Technology, Dolgoprudny, Moscow Region, Russia; 5 State Research Center for Applied Microbiology and Biotechnology, Obolensk, Moscow Region, Russia; 6 Higher Chemical College of the Russian Academy of Sciences, D. I. Mendeleev University of Chemical Technology of Russia, Moscow, Russia; 7 School of Life and Environmental Sciences, The University of Sydney, Sydney, Australia; Tianjin University, CHINA

## Abstract

*Acinetobacter baumannii* isolate NIPH 329 carries a novel capsular polysaccharide (CPS) gene cluster, designated KL46, that is closely related to the KL5 locus in *A*. *baumannii* isolate SDF but includes genes for synthesis of 5,7-diacetamido-3,5,7,9-tetradeoxy-l-*glycero*-l-*manno*-non-2-ulosonic (di-*N*-acetylpseudaminic) acid (Pse5Ac7Ac) instead of the corresponding D-*glycero*-D-*galacto* isomer (di-*N*-acetyllegionaminic acid) (Leg5Ac7Ac). In agreement with the genetic content of KL46, chemical studies of the K46 CPS produced by NIPH 329 revealed a branched tetrasaccharide repeat (K unit) with an overall structure the same as K5 from SDF but with â-Pse5Ac7Ac replacing α-Leg5Ac7Ac. As for K5, the K46 unit begins with d-Gal*p*NAc and includes α-d-Glc*p*NAc-(1→3)-d-Gal*p*NAc and α-d-Gal*p*-(1→6)-d-Glc*p*NAc linkages, formed by Gtr14 and Gtr15 glycosyltransferases, respectively. The Gtr94_K46_ glycosyltransferase, which is related to Gtr13_K5,_ links Pse5Ac7Ac to d-Gal*p* in the growing K unit via a â-(2→6) linkage. Nearly identical Wzy enzymes connect the K46 and K5 units via a α-D-Gal*p*NAc-(1→3)-α-D-Gal*p* linkage to form closely related CPSs. Both Pse5Ac7Ac in K46 and Leg5Ac7Ac in K5 are acetylated at O4 but no acetyltransferase gene is present in KL46 or KL5. Related acetyltransferases were found encoded in the NIPH 329 and SDF genomes, but not in other strains carrying an unacetylated Pse or Leg derivative in the CPS. The genes encoding the acetyltransferases were in different putative phage genomes. However, related acetyltransferases were rare among the >3000 publically available genome sequences.

## Introduction

Capsular polysaccharide (CPS) is an essential virulence determinant for the globally-significant bacterial pathogen, *Acinetobacter baumannii*, which has been listed by the World Health Organisation as the leading critical priority pathogen for therapeutics development due to the increasing prevalence of isolates with limited treatment options [1]. *A*. *baumannii* is a nosocomial pathogen that causes respiratory tract, wound, and urinary tract infections. As the outer-most antigenic component of the cell, the CPS has been exploited in various vaccine approaches and novel phage therapies. However, these strategies have challenges due to the extreme variation of the CPS structures in different isolates; more than 125 distinct CPS biosynthesis gene clusters have been identified (*J*.*J*. *Kenyon*, *unpublished data*) at the genomic K locus (KL) that directs the synthesis of the CPS [2]. The CPS of different isolates may have different sugar compositions and include different linkages between these sugars or between oligosaccharide repeats (K units) that make up the CPS polymers extending from the cell surface (e.g. [3–8]).

Given the importance of the CPS to development of alternate therapeutics, it is critical to examine the factors that give rise to structural variation. In the majority of cases studied to date, there has been perfect correlation between the genetic content at the K locus and CPS structure produced by the same isolate. When sugars were found to be modified by the addition of acetyl, acyl or pyruvyl residues, the gene for the transferase, acetyltransferase, pyruvyltransferase, etc., generally is present in the KL gene cluster [8–13]. However, in rare cases, genes identified elsewhere in the genome contribute to CPS synthesis or modification of the final CPS structure. For example, two different genomic islands (GIs), GI-1 and GI-2, have been described in the analysis of the *A*. *baumannii* K19 and K24 CPS, respectively [14, 15]. Both GIs carry a *wzy* gene, encoding the polymerase that links the K units together to form the long chain CPS, and the KL19 and KL24 gene clusters both lack a *wzy* candidate. GI-1 was further found to include an *atr* gene responsible for the modification of the K19 unit with an acetyl group [14].

Recently, the K5 CPS produced by *A*. *baumannii* isolate SDF was found to be 4-O-acetylated on the non-2-ulosonic acid residue, 5,7-diacetamido-3,5,7,9-tetradeoxy-D-*glycero*-D-*galacto*-non-2-ulosonic (di-N-acetyllegionaminic) acid (Leg5Ac7Ac). However, an acetyltransferase gene was not found in the KL5 gene cluster [5], suggesting that an unidentified gene located elsewhere in the genome may be involved. In this study, we examine the CPS structure produced by *A*. *baumannii* isolate NIPH 329 [16], which carries a novel KL gene cluster related to KL5, and correlate the structure with the whole genome sequence available.

## Materials and methods

### Bacterial strain and cultivation

*A*. *baumannii* NIPH 329 was isolated in the Czech Republic and obtained from Prof. Alexandr Nemec [16, 17]. Bacteria were cultivated in 2TY media for 16 h; cells were harvested by centrifugation (10,000×*g*, 20 min), washed with phosphate-buffered saline, suspended in a 7:3 (v/v) acetone-water mixture, precipitated by centrifugation, and dried on air.

### Isolation of the CPS

CPS was isolated by phenol-water extraction [18] of bacterial cells, the extract was dialyzed without layer separation and freed from insoluble contaminations by centrifugation. The resultant solution was treated with cold (4 °C) aqueous 50% CCl_3_CO_2_H; after centrifugation, the supernatant was dialyzed against distilled water and freeze-dried to give a CPS sample in a yield 9.5% of dry weight.

### Sugar analysis

A CPS sample (0.5 mg) was hydrolyzed with 2 M CF_3_CO_2_H (120 °C, 2 h). Monosaccharides were identified by GLC of the alditol acetates on a Maestro (Agilent 7820) chromatograph (Interlab, Russia) equipped with an HP-5ms column (0.32 mm × 30 m) and a temperature program of 160 °C (1 min) to 290 °C at 7 °C min^-1^.

### Mild acid hydrolysis of the CPS

A CPS sample (23 mg) was hydrolyzed with aqueous 2% HOAc (100 °C, 1 h), the products were fractionated by gel chromatography on a column (80 × 1.6 cm) of TSK HW-40 (S) in 1% HOAc using a differential refractometer (Knauer, Germany) for monitoring to give a modified polysaccharide (MPS) sample (7 mg) and Pse5Ac7Ac (2 mg).

### NMR spectroscopy

Samples were deuterium-exchanged by freeze-drying from 99.9% D_2_O and then examined as solution in 99.95% D_2_O. NMR spectra were recorded on a Bruker Avance II 600 MHz spectrometer (Germany) at 50 °C for CPS and MPS or 24 °C for Pse5Ac7Ac, using sodium 3-trimethylsilylpropanoate-2,2,3,3-d_4_ (δ_H_ 0, δ_C_ −1.6) as the internal reference. 2D NMR spectra were obtained using standard Bruker software, and Bruker TopSpin 2.1 program was used to acquire and process the NMR data. A mixing time of 100 and 150 ms was used in TOCSY and ROESY experiments, respectively.

### Bioinformatic analysis

The K locus sequence in the genome of NIPH 329 was extracted for analysis from WGS accession number APQY01000009 REGION: complement (584809–608726). Short reads for LUH5553 (KL90) and LUH5533 (KL7) were obtained from SRA accession numbers DRR006294 and DRR006297, and were assembled into contigs using the SPAdes assembly algorithm [19]. All novel gene clusters identified were annotated and characterised as described previously [2], and figures representing genetic sequences were constructed to scale using the Gene Construction Kit program. The sequence types (STs) of all isolates examined in this study were determined using the Pasteur MLST scheme (https://pubmlst.org/abaumannii/). Related KL gene clusters and phage-associated *atr* genes were identified using blastn (https://blast.ncbi.nlm.nih.gov/Blast.cgi). Genome or contig sequences of interest were examined for potential prophage regions using PHASTER (http://phaster.ca/) [20].

## Results

### KL gene cluster in the genome of *A*. *baumannii* NIPH 329

**Fig 1:**
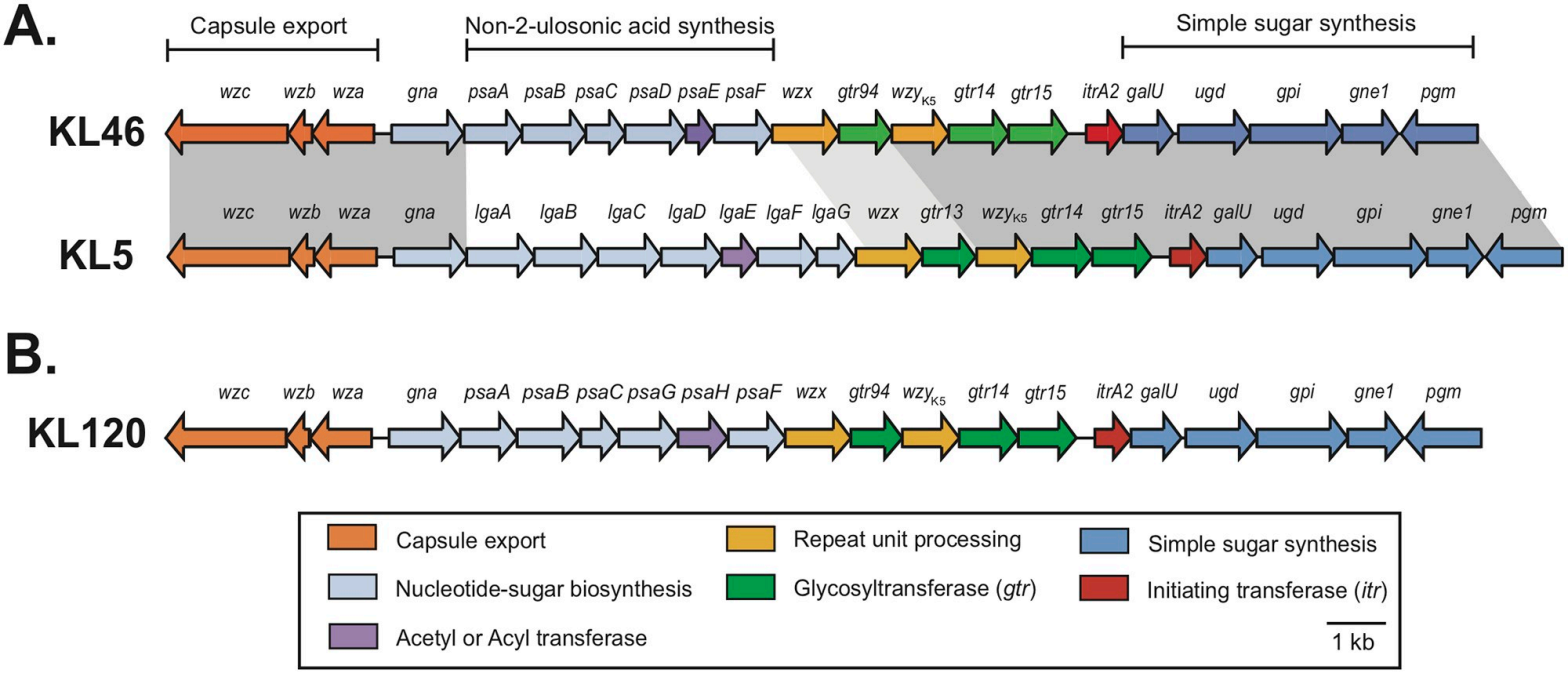
**A.** Comparison of the KL46 and KL5 capsular polysaccharide biosynthesis gene clusters of *A*. *baumannii* NIPH 329 and SDF, respectively. Modules of genes required for specific processes in CPS synthesis are indicated above. Dark shading between the gene clusters indicates >95% and light grey shading 80–95% nucleotide sequence identity. **B.** Organisation of the KL120 gene cluster that is similar to KL46. Colour scheme for the genes shown below indicates the functions of the encoded gene products. Figure is drawn to scale from GenBank accession numbers MK609549. 1 (KL46), CU468230.2 (KL5; coordinates 61721 to 89706), and LLCR01000062.1 (KL120; coordinates 12963 to 39906).

A novel CPS biosynthesis gene cluster was identified in the draft genome of *A*. *baumannii* isolate NIPH 329 (WGS accession number APQY01000009.1), annotated as described previously [2], and designated KL46 (GenBank accession number MK609549.1). The KL46 gene cluster (Fig 1A) has an arrangement that is typical for *A*. *baumannii* KL gene clusters [2] in that it includes a module of genes for CPS export (*wza-wzb-wzc*) and another one (*galU*, *ugd*, *gpi*, *gne1*, *pgm*) for synthesis of simple sugars on either side of a central region that is specific to the K46 CPS. The content of the KL46 gene cluster is similar overall to that of the KL5 gene cluster carried by *A*. *baumannii* isolate SDF (Fig 1A). However, in the central region, the KL46 gene cluster carries six genes, *psaABCDEF*, for synthesis of CMP-activated 5,7-di-*N*-acetylpseudaminic acid (Pse5Ac7Ac) [2] [21–23], whereas KL5 has *lgaABCDEFG* genes to make CMP-activated 5,7-di-*N*-acetyllegionaminic acid (Leg5Ac7Ac) [2][5].

KL46 has genes for three glycosyltransferases (*gtr94*, *gtr14* and *gtr15*) and the initiating transferase ItrA2 (*itrA2*) that transfers D-Gal*p*NAc-P from UDP-D-Gal*p*NAc to the UndP lipid carrier [21]. The combination of *gtr14*, *gtr15*, and *itrA2* genes were found previously in the KL5 gene cluster, and the ItrA2, Gtr14 and Gtr15 proteins from KL46 are 96–99% identical to those encoded by KL5. K5 includes D-Gal*p*NAc as the first sugar of the K unit. Gtr15 then adds D-Glc*p*NAc via an α-(1→3) linkage to the D-Gal*p*NAc sugar, and Gtr14 links a D-Gal*p* residue via an α-(1→6) linkage to the D-Glc*p*NAc [5]. Thus, it was expected that K46 will also include an α-D-Gal*p*-(1→6)-α-D-Glc*p*NAc-(1→3)-D-Gal*p*NAc segment. Gtr94_K46_ (GenPept accession number ENW42232.1) is 75% identical to Gtr13_K5_ (GenPept accession number CAO99488.1) that has previously been shown to link Leg5Ac7Ac to D-Gal*p* via an α-(2→6) linkage in biosynthesis of the K5 CPS [5]. Thus, it is likely that Gtr94_K46_ forms a â-Pse5Ac7Ac-(2→6)-D-Gal*p* linkage in K46.

The KL46 and KL5 gene clusters further include *wzy* genes that produce closely related proteins sharing 94% sequence identity. Wzy_K5_ has been shown to form an α-D-Gal*p*NAc-(1→3)-α-D-Gal*p* linkage between K5 units, and Wzy_K46_ should catalyse formation of the same linkage.

### Sugar compositions of the K46 CPS

Sugar analysis of the CPS_K46_ by GLC of the alditol acetates revealed galactose, glucosamine, and galactosamine. The d configuration of the monosaccharides was inferred from genetic data (see below). Further studies by NMR spectroscopy indicated that the CPS also contained Pse5Ac7Ac. The CPS was hydrolyzed under mild acidic conditions to give a modified Pse5Ac7Ac-lacking polysaccharide (MPS_K46_) and free Pse5Ac7Ac, which were isolated by GPC on Fractogel TSK HW-40. Pse5Ac7Ac was identified using NMR spectroscopy by comparison of the ^1^H and ^13^C NMR chemical shifts and ^3^*J*_H,H_ coupling constants with published data [24]. Formation of the MPS_K46_ upon mild acid hydrolysis indicates that Pse5Ac7Ac is a side-chain sugar.

### Structure elucidation of the K46 CPS

The ^1^H NMR and ^13^C NMR spectra of the MPS_K46_ showed signals for one residue each of β-GalNAc (**A**), α-GlcNAc (**B**), and α-Gal (**C**), all being in the pyranose form. The ^1^H NMR signals were assigned by H-1/H-2,3,4,5 correlations for GlcNAc and H-1/H-2,3,4 correlations for Gal and GalNAc in the 2D ^1^H,^1^H TOCSY spectrum combined with correlations between neighbouring protons within each monosaccharide residue in the 2D ^1^H,^1^H COSY spectrum. With the ^1^H NMR signals assigned, the ^13^C NMR spectrum of the MPS was assigned using a 2D ^1^H,^13^C HSQC experiment (Table 1).

**Table 1:** ^13^C and ^1^H NMR chemical shifts of the capsular polysaccharide (CPS) and a modified polysaccharide (MPS) from *A*. *baumannii* NIPH 329 (δ, ppm).

Residue	C-1	C-2	C-3	C-4	C-5	C-6	C-7	C-8	C-9
	*H-1*	*H-2*	*H-3 (3ax*,*3eq)*	*H-4*	*H-5*	*H-6 (6a*,*6b)*	*H-7*	*H-8*	*H-9*
CPS									
→3)-β-d-Gal*p*NAc-(1→	103.9	52.4	77.0	65.4	76.1	62.4			
**A**	*4*.*71*	*4*.*07*	*3*.*82*	*4*.*07*	*3*.*62*	*3*.*74*, *3*.*79*			
→6)-α-d-Glc*p*NAc-(1→	95.6	54.6	72.5	70.4	72.6	66.2			
**B**	*5*.*05*	*3*.*96*	*3*.*65*	*3*.*74*	*3*.*73*	*3*.*64*, *4*.*16*			
→3,6)-α-d-Gal*p*-(1→	99.7	68.7	80.6	70.5	70.5	65.2			
**C**	*4*.*97*	*3*.*92*	*3*.*95*	*4*.*21*	*4*.*05*	*3*.*58*, *3*.*95*			
β-Pse*p*4Ac5Ac7Ac-(2→	173.3	101.5	33.8	70.1	47.0	74.0	54.8	69.5	17.8
**D**			*1*.*76*, *2*.*50*	*4*.*90*	*4*.*30*	*4*.*00*	*4*.*07*	*4*.*13*	*1*.*19*
MPS									
→3)-β-d-Gal*p*NAc-(1→	103.8	52.3	76.9	65.4	76.1	62.3			
**A**	*4*.*71*	*4*.*07*	*3*.*81*	*4*.*07*	*3*.*62*	*3*.*73*, *3*.*79*			
→6)-α-d-Glc*p*NAc-(1→	95.5	54.6	72.5	70.4	72.6	66.2			
**B**	*5*.*05*	*3*.*96*	*3*.*65*	*3*.*72*	*3*.*73*	*3*.*68*, *4*.*09*			
→3)-α-d-Gal*p*-(1→	99.7	68.7	80.6	70.4	71.9	62.3			
**C**	*4*.*98*	*3*.*91*	*3*.*95*	*4*.*21*	*3*.*95*	*3*.*73*, *3*.*73*			

^1^H NMR chemical shifts are italicized. Chemical shifts for the N-acetyl groups are δ_H_ 1.92–2.05; δ_C_ 23.0–24.0 (Me) and 174.5–175.9 (CO); for the O-acetyl group δ_H_ 2.00; δ_C_ 21.7 (Me) and 174.1 (CO).

**Fig 2:**
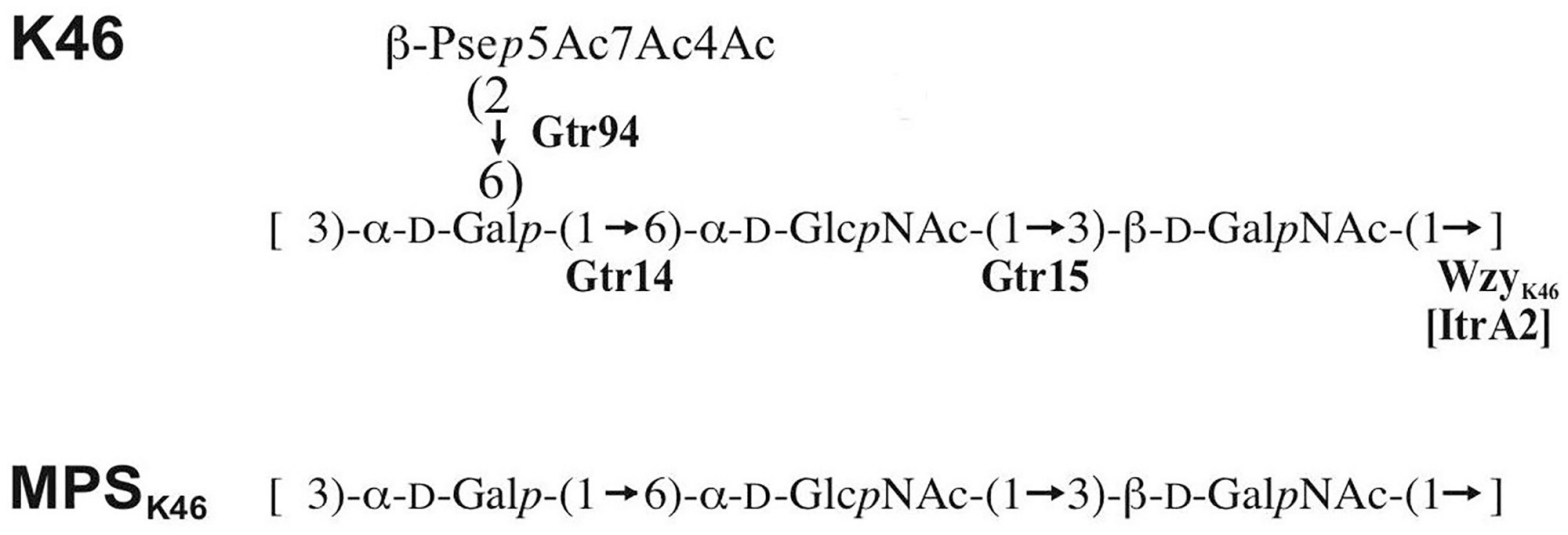
Structures of the capsular polysaccharides CPS_K46_ and the corresponding modified polysaccharide MPS_K46_ from *A*. *baumannii* NIPH 329.

The signals for C-3 of units **A** and **C** and C-6 of unit **B** at δ 76.9, 80.6, and 66.2 were shifted significantly downfield in the MPS_K46_, as compared with their positions in the corresponding non-substituted monosaccharides at δ 72.4, 70.4, and 61.9, respectively [25]. These displacements are characteristic of signals for linkage carbons and showed that the MPS_K46_ is linear and defined the glycosylation pattern in the K unit (Fig 2).

The order of the monosaccharide residues in the MPS_K46_ shown in Fig 2 was determined by the 2D ^1^H,^1^H ROESY spectrum, which showed α-Gal H-1/α-GlcNAc H-6a,6b, α-GlcNAc H-1/β-GalNAc H-3, and β-GalNAc H-1/α-Gal H-3 correlations at δ 4.98/3.68, 5.05/3.81, and 4.71/3.95, respectively. It was confirmed by α-Gal H-1/α-GlcNAc C-6, α-GlcNAc H-1/β-GalNAc H-3, and β-GalNAc H-1/α-Gal C-3 correlations at δ 4.98/66.2, 5.05/76.9, and 4.71/80.6, respectively, which were observed in the ^1^H,^13^C HMBC spectrum of the MPS_K46_.

**Fig 3:**
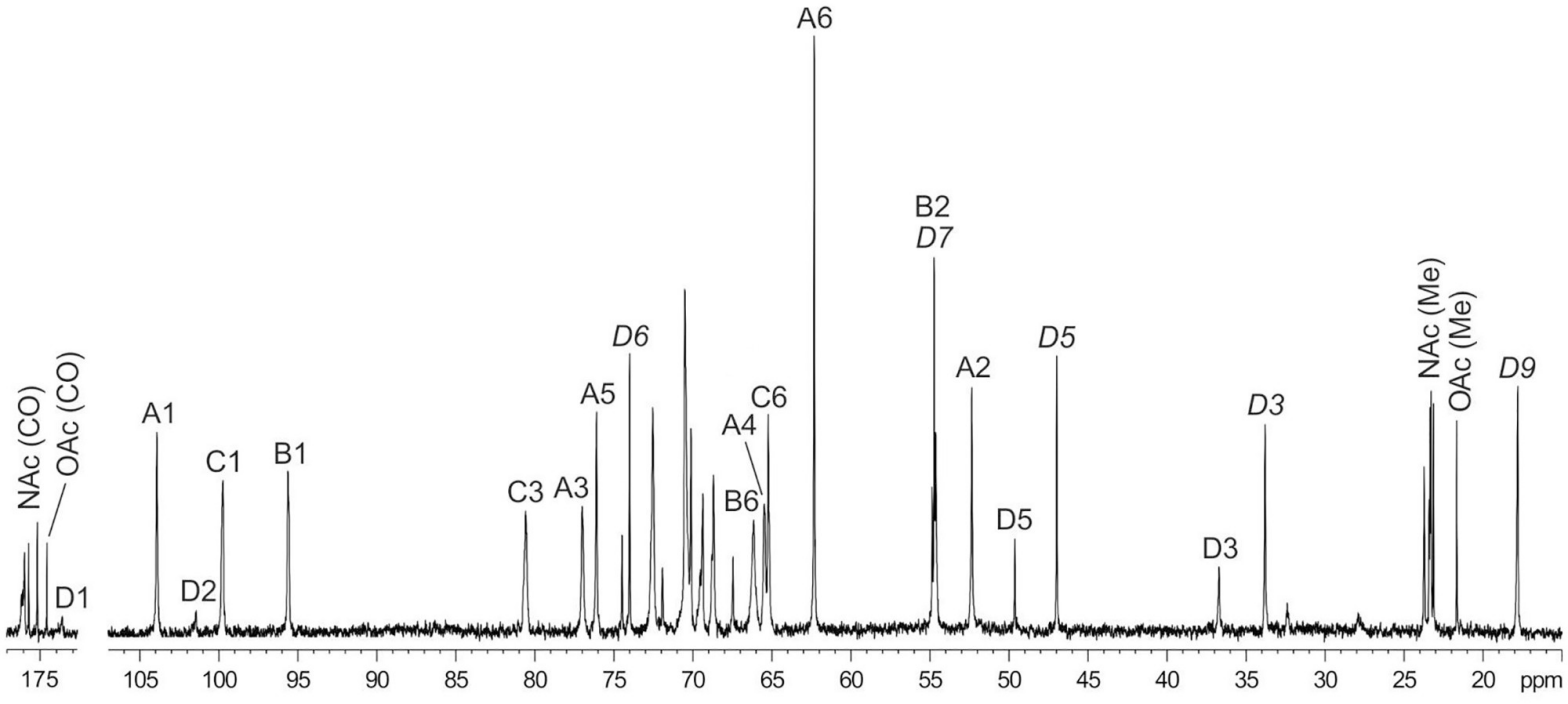
^13^C NMR spectrum of the capsular polysaccharide CPS_K46_ from *A*. *baumannii* NIPH 329. Numbers refer to carbons in sugar residues denoted by letters as shown in Table 1. Signals of Pse4Ac5Ac7Ac are annotated in italics.

The ^1^H NMR and ^13^C NMR (Fig 3) spectra of the CPS_K46_ showed signals for the same three monosaccharide residues (units **A-C**) as present in the MPS_K46_ and, in addition, those for β-Pse*p*5Ac7Ac (unit **D**), which was identified as described [26]. Particularly, the axial orientation of the carboxyl group, i.e. the β configuration of Pse, was inferred from a relatively large difference of 0.87 ppm between the chemical shifts of H-3ax and H-3eq in the ^1^H NMR spectrum [24]. The anomeric configuration of Pse was confirmed by the C-6 chemical shift of δ 74.6–74.7 (compare published data [24] δ 71.4 and 74.3 for α- and β-Pse*p*5Ac7Ac, respectively).

In the ^13^C NMR spectrum of the CPS_K46_, the signal for C-6 of α-Gal*p* was shifted downfield to δ 65.2, as compared with its position at δ 62.3 in the spectrum of the MPS_K46_; hence β-Pse*p*5Ac7Ac was attached at position 6 of α-Gal*p*. The terminal position of pseudaminic acid in the side chain was confirmed by the results of mild acid hydrolysis of the CPS_K46_ giving rise to MPS_K46_ (see above) and a similarity of the ^13^C NMR chemical shifts of unit **D** (Table 1) with published data for free β-Pse*p*5Ac7Ac [24].

### K46 is O acetylated

The NMR spectra of the CPS_K46_ showed signals for an O-acetyl group (δ_H_ 2.00, δ_C_ 21.7 and 174.1). In the ^13^C NMR spectrum of the CPS_K46_, a major part of the signal for C-4 of Pse5Ac7Ac was shifted downfield to δ 70.1, and those for C-3 and C-5 were shifted upfield to δ 33.8 and 47.0, as compared with their positions in the non-O-acetylated Pse5Ac7Ac at δ 67.5, 36.8, and 49.7, respectively. These displacements were evidently due to 4-O-acetylation of Pse5Ac7Ac and thus defined the position of the O-acetyl groups in the CPS_K46_. As judged by the ratio of integral intensities of the signals for Pse4Ac5Ac7Ac and Pse5Ac7Ac, the degree of O-acetylation is ~75%.

### Relationship of the CPS_K46_ structure to the KL46 gene cluster sequence

The CPS produced by *A*. *baumannii* isolate NIPH 329 (Fig 2), consists of Pse4Ac5Ac7Ac, D-Gal*p*, D-Glc*p*NAc and D-Gal*p*NAc sugars, as expected. Given the presence of *itrA2* in the KL46 gene cluster, D-Gal*p*NAc is drawn as the first sugar of the K unit in Fig 2. Like Wzy_K5_, Wzy_K46_ would therefore catalyse formation of a â-D-Gal*p*NAc-(1→3)-D-Gal*p* linkage between K units in the K46 CPS. The K46 structure further includes the expected α-D-Gal*p*-(1→6)-â-D-Glc*p*NAc-(1→3)-D-Gal*p*NAc segment, and formation of these linkages is catalysed by Gtr14_K46_ and Gtr15_K46_, respectively. Thus, Gtr94_K46_ would be responsible for the addition of the final Pse5Ac7Ac residue to D-Gal*p* via a â-(2→6) linkage as predicted (Fig 2). The K46 structure is therefore largely consistent with the content of the KL46 gene cluster. However, the only *atr* gene in KL46 lies in the *psa* gene cluster (purple in Fig 1) and is responsible for the acetylation of Pse at N5 and N7. Hence, a candidate *atr* gene encoding an acetyltransferase (Atr) for the addition of the 4-O-acetyl group to ~75% of the Pse5Ac7Ac residues in the K46 CPS was not found at the KL46 locus.

### Identification of an acetyltransferase for 4-O-acetylation of Pse5Ac7Ac in K46

**Fig 4:**
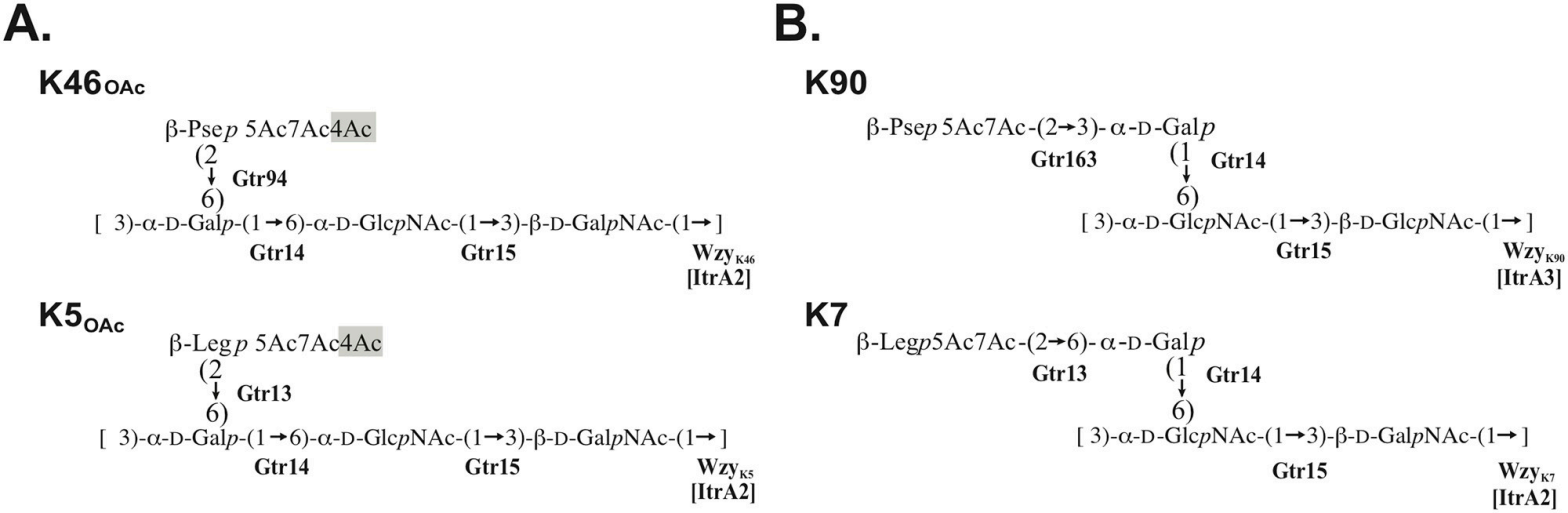
**A.** CPS structures NIPH329 (this work) and SDF [5] with 4-O-acetylation of the non-2-ulosonic acid residue. **B.** Related CPS structures of *A*. *baumannii* LUH5553 [28] and LUH5533 [27] without 4-O-acetylation of the non-2-ulosonic acid residue. Glycosyltransferases, polymerases and acetyltrasferases are indicated in bold next the linkage they are assigned to. The 4-*O*-acetyl group is shaded in grey.

The acetylation pattern in K46 is similar to that of the K5 CPS produced by *A*. *baumannii* isolate SDF [5], which includes Leg5Ac7Ac residues that are also 4-O-acetylated (Fig 4A). Similarly, the KL5 gene cluster does not include a suitable *atr* gene (Fig 1). This suggests that an *atr* gene located elsewhere in the genome may be involved in the acetylation of the non-2-ulosonic acids in the K46 and K5 CPS units. The potential *atr* gene(s) should be present in the genomes of NIPH 329 and SDF but absent in strains with related but unacetylated CPS structures, such as K7 and K90 CPS produced respectively by *A*. *baumannii* isolates LUH5533 [27] and LUH5553 [28].

Assuming that the candidate Atr belongs to an established protein family, the draft genome sequence of isolate NIPH 329 was investigated for CDS features annotated as either ‘acetyltranferase’, ‘acyltranferase’, or ‘GNAT family acetytransferase’. A total of 27 gene candidates were identified (S1 Table), and each of these were further assessed to identify related genes present in the SDF genome sequence (GenBank accession number CU468230.2) but absent from the genome sequences of isolates LUH5553 and LUH5533 assembled here from available short read data (SRA numbers DRR006294 and DRR006297, respectively). Only one Atr candidate (locus tag F919_03690) encoded by the NIPH 329 genome sequence, designated Atr29 (GenPept accession number ENW41154.1), was related (80% identical) to an Atr, designated Atr30 (GenPept accession number CAP00403.1), encoded by the SDF genome, but Atr29 and Atr30 had no relative in the assembled genome sequences of LUH5533 (KL7) and LUH5553 (KL90).

To further validate the conclusion that Atr29 and Atr30 are responsible for the observed 4-O-acetylation, the genomes of all isolates producing CPS structures containing a derivative of either pseudaminic acid or legionaminic acid were examined (Table 2). None encoded a homologue of Atr29/Atr30. Furthermore, relatives of the *atr* gene were not widely distributed in *A*. *baumannii*, being found in only a small number of complete and draft genome sequences.

**Table 2:** Pseudaminic acid and legionaminic acid derivatives.

K Type	Reference	K locus genes^2^	Phage *atr*
**Pse5Ac7Ac**			
K2	[21]	*psaABC****DE****F*	-
K6	[22]	*psaABC****DE****F*	-
K16	[8]	*psaABC****DE****F*	-
K33	[29]	*psaABC****DE****F*	-
K90	[28]	*psaABC****DE****F*	-
**Pse5Ac7Ac4OAc**			
K46	This study	*psaABC****DE****F*	*atr29*
**Pse5Ac7R**^1^			
K42	[30]	*psaABC****GH****F*	-
K93	[26]	*psaABC****GH****F*	-
**Leg5Ac7Ac**			
K7	[27]	*lgaABC****DE****FG*	-
K54	Unpublished	*lgaABC****DE****FG*	-
**Leg5Ac7Ac4OAc**			
K5	[5]	*lgaABC****DE****FG*	*atr30*
**Leg5Ac7R**^1^			
K27	[3]	*lgaABC****HI****FG*	-
K44	[3]	*lgaABC****HI****FG*	-
K63	[31]	*lgaABC****HI****FG*	-
K8	Unpublished	*lgaABC****HI****FG*	-

^1^ R is either Ac or 3-hydroxybutanoyl (Hb)

^2^ Bold face genes determine the acylation/acetylation pattern of the sugar

### The *atr29* and *atr30* genes are in phage genomes

The genetic context of the candidate *atr29* gene was examined in the NIPH 329 genome sequence, and the genes located either side of *atr29* were found to encode proteins related to phage proteins. The specific contig of the NIPH 329 draft genome containing the *atr29* gene (WGS accession number APQY01000013.1) was therefore subjected to PHASTER analysis, which identified 44 of 59 encoded proteins of phage origin and *attL* and *attR* sites at both ends of the 42249 bp contig. As no adjacent chromosomal sequence was present, it was not possible to locate the position of the prophage in the NIPH 329 genome.

The SDF genome was also analysed using PHASTER, and the *atr30* gene was found close to a potential phage region of ~23.6 kb (GenBank accession number CU468230.2; coordinates 932745–956361) at ~0.8 Mb from the K locus in the SDF chromosome (Table 3). However, this prophage sequence shared no significant identity with that in NIPH 329, suggesting that there are multiple members of the *atr29/atr30* gene family, each in a different phage genome.

**Table 3:** *A*. *baumannii* genome sequences with phage genomes carrying an *atr* gene for 4-O-acetylation of the CPS.

Strain	GenBank accession number	Phage match	Coordinates of phage genome	Coordinates of *atr29/atr30*	GenPept accession number of Atr29/Atr30	Amino acid sequence identity^a^
NIPH329	APQY01000013.1	PHAGE_Acinet_vB_AbaS_TRS1_NC_031098(28) [incomplete]	1-42232	39593–40639 *(atr29)*	ENW41154.1	100%
ABBL011	LLCR01000058.1	PHAGE_Acinet_vB_AbaS_TRS1_NC_031098(27) [incomplete]	97376-149013	141964-143010*(atr29)*	KRI30500.1	99%
ARLG1935	NGIJ01000016.1	PHAGE_Acinet_Bphi_B1251_NC_019541(11) [questionable]	145514-178780	145062–146108 *(atr29)*	(CAT67_11335)^b^	92%
SDF	CU468230.2	PHAGE_Entero_mEp235 [questionable]	932745–956361	959212–960231 *(atr30)*	CAP00403.1	80%

^a^ Amino acid sequence identity is to GenPept accession number ENW41154.1 from NIPH329.

^b^ Translated into amino acid sequence from locus tag indicated in parentheses.

### *A*. *baumannii* isolates carrying related candidate *atr* genes

A homologue of Atr29 with 99% amino acid (aa) sequence identity was encoded by a gene carried by *A*. *baumannii* isolate ABBL011 (WGS accession number LLCR01000058.1), and was also located in a phage-associated sequence region. However, the surrounding sequence was not closely related to the *atr29-*associated phage genome in NIPH 329. ABBL011 was found to carry a new KL variant, KL120 (GenBank accession number LLCR01000062.1; coordinates 12963 to 39906), a close relative of KL46 in which two genes in the *psa* gene cluster (*psaG/H* in KL120 and *psaD/E* in KL46) have been replaced (Fig 1B). The presence of *psaG/H* predicts the synthesis of Pse5Ac7Hb (where Hb is 3-hydroxybutanoyl), thus it is likely that KL120 directs the synthesis of a CPS similar to K46 but with Pse5Ac7Hb replacing Pse5Ac7Ac. ABBL011 belongs to ST6 in the Pasteur MLST scheme, whereas NIPH 329 belongs to the unrelated ST11.

A homologue of Atr29 with 92% amino acid (aa) sequence identity was also encoded by a phage-like sequence in *A*. *baumannii* isolate ARLG1935 (WGS accession numbers NGIJ01000016.1) but this phage genome was again not related to that in NIPH 329 (Table 3). This isolate also carries KL46 (WGS accession number NGIJ01000019.1; coordinates 31657 to 58229) and, like NIPH 329, belongs to ST11 indicating that more than one phage can bring in an *atr* gene that has the potential to modify the CPS.

### 4-O-Acetylation of the K46 is not universal

As close homologues of the *atr* gene product encoded by NIPH329 and SDF, which produce CPS with 4-O-acetylation of a non-2-ulosonic acid, are found in isolates ARLG1935 and ABBL011 that carry related KL gene clusters, it is likely that Atr29 and Atr30 also perform 4-O-acetylation of Pse and Leg residues in these CPSs. However, the KL46 gene cluster was further identified in the genome sequence of *A*. *baumannii* isolate TG22162 (WGS accession number RFCR01000020.1; coordinates 14854 to 41423). The *atr29* gene was not found in the genome sequence of isolate TG22162 suggesting that 4-O-acetylation does not occur in all isolates producing a K46 CPS structure.

## Discussion

The K46 CPS structure elucidated in this study includes a 4-O-acetylated variant of pseudaminic acid attached as a side chain to a á-D-Gal*p*-(1→6)-â-D-Glc*p*NAc-(1→3)-D-Gal*p*NAc backbone via a â-(2→6) linkage to D-Gal*p*. Formation of all these linkages catalyzed by the glycosyltransferases encoded by the KL46 gene cluster could be assigned due to the close relationship to those encoded in the KL5 gene cluster. Their roles were previously assigned via comparison of the K5 [5] and K7 [27] CPS structures produced by isolates carrying the KL5 and KL7 gene clusters, a closely related pair that differ only in a segment that includes the *wzy* gene [2]. KL46 and KL5 are also a closely related pair of gene clusters that differ only in a central segment that includes the genes for synthesis of the non-2-ulosonic acid side-branch (Fig 1).

Though the K46 structure is largely consistent with the content of the KL46 gene cluster, the 4-O*-*acetylation of the Pse5Ac7Ac residue could not be explained by any gene located at the K locus, and a similar situation was observed previously for K5 [5]. In this study, we identified a potential *atr* candidate, *atr29*, in the genome of NIPH 329 in a region likely to be of phage origin, though it could not be placed in the chromosome. A homologue of the *atr29* gene, *atr30*, was also found encoded by a potential phage genome in the SDF chromosome and the K5 CPS of SDF is also 4-O-acetylated. We also identified a further two phage-associated Atr29 homologues in the genomes of two other isolates that carry KL gene clusters with Pse5Ac7Ac biosynthesis genes. Weaker homologues of 70–80% aa sequence identity with >96% sequence coverage were also found encoded by a further 17 *A*. *baumannii* genome sequences (data not shown). However, the precise role of Atr29/Atr30 and its homologues in CPS synthesis remains to be confirmed, and in this regard determination of the structure of the CPS produced by TG22162, which also carries KL46 but does not encode any Atr29 homologue may be valuable.

The discovery of polysaccharide genes in prophage sequences, in particular acetyltransferase genes that contribute to surface polysaccharide variation in bacteria, has been reported in other Gram-negative bacteria including *Salmonella* [32, 33], *Shigella* [34, 35] and *Pseudomonas* [36]. Previously, it has been suggested that the incorporation of polysaccharide biosynthesis genes into phage genomes confers a potential fitness advantage for the phage, via the alteration of the CPS structure to prevent other phage that recognise the same structure from infecting the cell [35]. However, the finding of CPS genes in phage sequence demonstrates that some CPS biosynthesis genes are able to transfer between cells via mechanisms other than by homologous recombination at the K locus. Interestingly, both GI-1 and GI-2 regions with CPS genes were found to be flanked by direct repeats in *A*. *baumannii* genomes, suggesting that these islands had also inserted into the chromosome and may also be mobile. However, the exact mechanism of this transfer is still unknown. These additional evolutionary transfer mechanisms could assist with the rapid alteration of the CPS structure, and this could prevent recognition by antibodies or phage-encoded hydrolases. This would in turn potentially complicate vaccine and phage therapies used for treatment of *A*. *baumannii* infections that target specific CPS structures.

## Supporting information

S1 Table Annotated acetyltransferases identified in the genome sequence of *A*. *baumannii* NIPH 329. (DOCX)
