# Supplementary material for: The K46 and K5 capsular polysaccharides produced by Acinetobacter baumannii NIPH 329 and SDF have related structures and the side-chain non-ulosonic acids are 4-O-acetylated by phage-encoded O-acetyltransferases
Source: PLoS One. 2019 Jun 20;14(6):e0218461. doi: 10.1371/journal.pone.0218461 (PMC6586298; doi:10.1371/journal.pone.0218461)
Supplement: S1 Table — (DOCX) [file pone.0218461.s001.docx]

**Table S1. Annotated acetyltransferases identified in the genome sequence of *A. baumannii* NIPH 329**

| **Contig** | **WGS accession number** | **Contig size** | **Annotation** | **GenPept accession number** |
| --- | --- | --- | --- | --- |
| Acinetobacter baumannii NIPH 329 acMab-supercont1.1 | NZ_KB849868.1 | 530787 bp | GNAT family N-acetyltransferase | WP_001123235.1 |
|  |  |  | GNAT family N-acetyltransferase | WP_000469967.1 |
|  |  |  | acyltransferase | WP_005122241.1 |
| Acinetobacter baumannii NIPH 329 acMab-supercont1.2 | NZ_KB849869.1 | 259697 bp | GNAT family N-acetyltransferase | WP_002061438.1 |
| Acinetobacter baumannii NIPH 329 acMab-supercont1.3 | NZ_KB849870.1 | 388924 bp | - | - |
| Acinetobacter baumannii NIPH 329 acMab-supercont1.4 | NZ_KB849871.1 | 2662207 bp | acyltransferase | WP_000713973.1 |
|  |  |  | acyltransferase | WP_000100967.1 |
|  |  |  | N-acetyltransferase | WP_002132144.1 |
|  |  |  | GNAT family N-acetyltransferase | WP_001166565.1 |
|  |  |  | GNAT family N-acetyltransferase | WP_000637089.1 |
|  |  |  | N-acetyltransferase | WP_005123778.1 |
|  |  |  | N-acetyltransferase | WP_005123906.1 |
|  |  |  | GNAT family N-acetyltransferase | WP_002132998.1 |
|  |  |  | GNAT family N-acetyltransferase | WP_005124012.1 |
|  |  |  | N-acetyltransferase | WP_000956973.1 |
|  |  |  | N-acetyltransferase | WP_001109741.1 |
|  |  |  | N-acetyltransferase | WP_000060331.1 |
|  |  |  | N-acetyltransferase | WP_005124234.1 |
|  |  |  | GNAT family N-acetyltransferase | WP_005124323.1 |
|  |  |  | N-acetyltransferase | WP_002163310.1 |
|  |  |  | GNAT family N-acetyltransferase | WP_005124378.1 |
|  |  |  | GNAT family N-acetyltransferase | WP_001026238.1 |
|  |  |  | GNAT family N-acetyltransferase | WP_000890283.1 |
|  |  |  | GNAT family N-acetyltransferase | WP_005124638.1 |
|  |  |  | GNAT family N-acetyltransferase | WP_000803551.1 |
|  |  |  | GNAT family N-acetyltransferase | WP_005124865.1 |
|  |  |  | GNAT family N-acetyltransferase | WP_005124885.1 |
| Acinetobacter baumannii NIPH 329 acMab-supercont1.5 | NZ_KB849872.1 | 7946 bp | - | - |
| Acinetobacter baumannii NIPH 329 acMab-supercont1.6 | NZ_KB849873.1 | 10014 bp | - | - |
| Acinetobacter baumannii NIPH 329 acMab-supercont1.7 | NZ_KB849874.1 | 42249 bp | acyltransferase | WP_005127322.1 |
| Acinetobacter baumannii NIPH 329 acMab-supercont1.8 | NZ_KB849875.1 | 103924 bp | - | - |
